# *Euphorbia dracunculoides* L. abrogates carbon tetrachloride induced liver and DNA damage in rats

**DOI:** 10.1186/s12906-017-1744-x

**Published:** 2017-04-20

**Authors:** Riffat Batool, Muhammad Rashid Khan, Muhammad Majid

**Affiliations:** 1 Department of Biochemistry, Faculty of Biological Sciences, Quaid-i-Azam University, Islamabad, 45320 Pakistan; 2 Department of Pharmacy, Faculty of Biological Sciences, Quaid-i-Azam University, Islamabad, 45320 Pakistan

**Keywords:** *Euphorbia Dracunculoides*, Oxidative stress, Lipid peroxidation, Genotoxicity

## Abstract

**Background:**

Evaluation of *Euphorbia dracunculoides* of family Euphorbiaceae during previous studies had established the in vitro antioxidant and in vivo anti-inflammatory activities. The plant is used by the local communities of Pakistan for various disorders including rheumatism and edema. In this investigation we have evaluated the hepatoprotective effects against CCl_4_ induced toxicity in rat.

**Methods:**

Dry powder of the aerial parts of *E. dracunculoides* was extracted with 95% methanol to get the extract (EDME). To investigate the hepatoprotective effects of EDME the Sprague-Dawley male rats were divided in to 8 groups with 6 rats in each. Group I and II were the normal and vehicle treated while the Groups III-VI were injected intraperitoneally with 1 ml of CCl_4_ (30% in olive oil). Rats of Group IV were orally administered with silymarin (50 mg/kg) while the Group V and VI with 200 mg/kg and 400 mg/kg of EDME, respectively. Animals of Group VII (200 mg/kg) and VIII (400 mg/kg) were treated with EDME alone. The treatments were given thrice a week for 4 weeks. Effects of EDME were evaluated for the protective effects against oxidative stress and genotoxicity induced with CCl_4_ in liver of rat.

**Results:**

Analysis of serum indicated significant (*p* < 0.05) rise in the level of aspartate transaminase (AST), alanine transaminase (ALT), alkaline phosphatase (ALP) and globulin whereas decrease was recorded for the total protein and albumin in CCl_4_ treated rats. In liver tissues the activity level of catalase (CAT), peroxidase (POD), superoxide dismutase (SOD), glutathione-S-transferase (GST), glutathione (GSH) was decreased while the level of lipid peroxides; thiobarbituric acid reactant substances (TBARS), nitrite and hydrogen peroxide increased in CCl_4_ treated rats as compared to the control group. Histopathological injuries and DNA damages were recorded in liver of rat with CCl_4_ treatment. However, co-administration of EDME, dose dependently, ameliorated the CCl_4_-induced hepatic toxicity in these parameters.

**Conclusions:**

These results suggested that the phyto-constituents of EDME were able to ameliorate the oxidative stress induced with CCl_4_ and can be a useful therapeutic agent for oxidative stress related disorders.

## Background

The reactive oxygen species (ROS) and reactive nitrogen species (RNS) are produced as byproduct of metabolic pathways. The reactive species include hydrogen peroxide (H_2_O_2_), hydroxyl radical (OH), nitric oxide (NO) and superoxide anion (O_2_^−^). Production of such species in trivial amounts is required for normal physiological functions but surplus yield can cause oxidative stress. Drugs, chemicals, pollutant and radiations are the common sources for the generation of free radicals which cause damages to cellular organization by affecting membranes, proteins, lipids as well as nucleic acids [1, 2]. Such effects have been observed in pathological conditions like diabetes mellitus, Alzheimer’s disease, liver cirrhosis, rheumatic arthritis, cancer and multiple sclerosis [3, 4].

Carbon tetrachloride (CCl_4_) can cause hepatotoxicity by producing oxidative stress which leads to steatosis and centrilobular necrosis and its prolonged administration produces chronic liver injury which leads to hepatic fibrosis [5]. In recent years, antioxidants have been isolated from plants to scavenge free radicals. Within the antioxidant compounds, flavonoids and phenolic acids have the considerable antioxidative potential [6]. The phenolic compounds show the antioxidant activity because of the presence of hydroxyl group in conjugated ring in their structure, and hence these can prevent free radical mediated diseases. Antioxidants protect the tissues by scavenging ROS [7], singlet oxygen [8] and superoxide anion [9].

Euphorbiaceae is one of the most varied family, ranges from the herb, shrub and tall tree [10]. The largest among the spurge family is the genus *Euphorbia* covering over 2000 species. Most of the species of this genus are used conventionally in Chinese medicinal system for curing skin disorders, edemas etc. [11]. *Euphorbia dracunculoides* Lam. a short-lived perennial herb attaining height up to 10–40 cm is dispersed in valleys, riverbanks, roadsides of filthy areas in Southwest Asia, South Europe and North Africa. It has been used as a traditional medicine in India for its diuretic and laxative effects [12]. A decoction of whole plant is effective in killing lice when applied to the body of cattle [13]. Fruits are known for their potential to exterminate warts from the skin [14]. Leaf paste mixed with powder of the black pepper and ghee is useful in snake bite whereas leaf powder is used in epilepsy [15]. Hepatoprotective effects of *E. nematocypha* have been investigated on hepatocyte cell lines [16] whereas in vivo investigation of *E. neriifolia* against CCl_4_ induced hepatic damages in rat has been reported by Bigoniya and Rana [17]. Aamir et al. [18] reported the hepatoprotective potential of *E. thymifolia* against CCl_4_ induced hepatic damages in rat.

The medicinal propensity can be assessed by initial qualitative phytochemical screening of plants. Evalaution of *E. dracunculoides* confirmed the presence of coumarins, terpenoids, tannins, flavonoids, phenols, saponins, alkaloids and betacyanin. GC-MS analysis of *n*-hexane extract revealed the presence of 30 chemical constituents. Analysis of aqueous extract through HPLC confirmed the presence of four compounds i.e. catechin, rutin, myricetin and caffeic acid. Catechin is a polyphenol and has excellent antioxidant potential against free radicals and provides protection against inflammation, neurological disorders and apoptosis. Rutin is a well reputed secondary metabolite of plants with admirable anti-inflammatory, hepatoprotective and antioxidant activities. Various antioxidant assays exhibited significant correlation with that of total phenolic and total flavonoids contents [19]. Non-toxic attitude with profound anti-inflammatory and antioxidant assertiveness of *E. dracunculoides* make it a strong candidate for in vivo evaluation against oxidative stress induced by CCl_4_ in rat. In this concern, activity of hepatic antioxidant enzymes and biochemical investigation of serum was executed. Also to check protective potential of the extract at genetic level, comet assay was conducted.

## Methods

### Plant collection

Plant material was collected in April 2015 from District Lakki Marwat, Pakistan. Then recognized by its native name and confirmed by Dr. Rizwana Aleem Qureshi, Department of Plant Sciences, Quaid-i-Azam University, Islamabad. Voucher specimen, with Accession No. 127962 was deposited at the Herbarium of Pakistan, Quaid-i-Azam, University, Islamabad, Pakistan**.**

### Preparation of extract

The aerial part of the plant was washed to get free of dust constituents and shade dried for 3 weeks at room temperature (20–25 °C). The fully dried plant was ground with an electric grinder. The powder (1.2 kg) was macerated twice at room temperature with 3 l of 95% methanol for 48 h. Filtrate was dried in rotary vacuum evaporator at 40 °C to obtain crude methanol extract of *E. dracunculoides* (EDME).

### Acute toxicity study

Acute toxicity of EDME was investigated on Sprague-Dawley rats including females and males. The rats in each group were three (females 2; male 1) and were orally administered during morning (fasting conditions) with EDME at 50, 250, 500, 1000, 2000, 3000, 4000 mg/kg. Saline (10 ml/kg) was administered to the control rats. The animals were examined once daily for 14 days for mortality, behavioral pattern (lethargy, sleep, salivation), changes in physical appearance, injury, pain, and signs of illness.

### Experimental design

The protocol of Shyu et al. [20] was trailed to do this experiment. Forty eight (48) male Sprague Dawley rats (180–200 g) were used for experiment. Guidelines of National Institute of Health, Islamabad were strictly followed in order to conduct experiment effectively. The designed protocol was ratified (Bch#0265) by the Ethical Committee of Quaid-i-Azam University, Islamabad, Pakistan. Animals were kept at room temperature with a dark/light 12 h phase in ordinary cages. Animals were properly fed on usual laboratory feed and water. The arbitrarily distribution of rats was done in eight different groups and each group was treated with its respective dose or combination of doses in calculated amount. Group I was remained untreated and only standard food supply was offered. Group II labeled as vehicle control was orally administered with 10% DMSO in olive oil (1 ml/kg). Rats of Group III were intraperitoneally injected with 1 ml/kg of 30% CCl_4_ (in olive oil). Animals of Group IV were administered with 1 ml/kg of 30% CCl_4_ (in olive oil) + silymarin dissolved in DMSO (50 mg/kg). Rats of group V and group VI were treated with 1 ml/kg of 30% CCl_4_ (in olive oil) + EDME dissolved in DMSO (200 mg/kg and 400 mg/kg, respectively) whereas Group VII and VIII were administered with EDME alone dissolved in DMSO (200 mg/kg and 400 mg/kg, respectively). The treatments of CCl_4_ and EDME were given in the morning on alternate days thrice a week for 4 weeks. After last treatment rats were unfed for 24 h and were euthanized after sedation with chloroform. Blood was collected for serum analysis. The liver was excised and placed in saline solution. A part of the liver was stored in liquid nitrogen for enzymatic and biochemical studies while the other portion was stored in 10% formalin solution histopathological studies.

### Serum analysis

For assessment of liver function tests, serum mockups were analyzed for AST, ALT, ALP, albumin, globulin and total protein by means of AMP diagnostic kits (Graz, Austria) according to the instructions of the manufacturer.

### Biochemical analysis

#### Assessment of antioxidative profile

Tissues of liver from various groups were homogenized in 10X prepared by the addition of 100 mM potassium phosphate buffer having 1 mM EDTA (pH 7.4). Centrifugation of homogenate was done at 12000×g at 4 °C for 30 min. In the supernatant antioxidant enzyme assays which are listed below were performed.

#### Catalase assay (CAT)

Catalase activity was measured by the procedure of Chance and Maehly [21]. The reaction mixture for the catalase assay contained 2500 μl of phosphate buffer (pH 5.0; 50 mM), 100 μl of the supernatant and 400 μl of H_2_O_2_ (5.9 mM). Change in absorbance was determined at 240 nm after a minute. One unit catalase activity was demarcated as an absorbance variation of 0.01 units per minute.

#### Peroxidase assay (POD)

Peroxidase commotion was measured by the Chance and Maehly [21] method. Reaction mixture of peroxidase assay contained 2500 μl of phosphate buffer, (pH 5.0; 50 mM), 150 μl of guaiacol (20 mM), 300 μl of H_2_O_2_ (50 mM) and 1000 μl of the supernatant. Change in absorbance after a minute was recorded at 470 nm. One unit peroxidase activity was described as an absorbance change of 0.01 units per minute.

#### Superoxide dismutase, assay (SOD)

SOD assay was performed by following the Kakkar et al. [22] method. The reaction mixture contained 100 μl of phenazine methosulphate (186 μM), 1200 μl of sodium pyrophosphate buffer (pH 7.0; 0.052 mM) and 300 μl of supernatant obtained from liver homogenate which was incorporated to reaction amalgam after centrifugation at 1500 rpm for 10 min and then at 10000 rpm for 15 min. The reaction was started by adding 0.2 ml of NADH (780 μM) and then stopped on adding 1000 μl of glacial acetic acid after 1 min. Absorbance was recorded at 560 nm and results were articulated in units/mg protein.

#### Glutathione-S-transferase assay (GST)

GST was assessed by following Habig et al. [23] method. The reaction assortment for this assay contained 1475 μl of phosphate buffer (pH 6.5; 0.1 M), 200 μl of reduced glutathione (1 mM), 25 μl of 1 mM 1-chloro-2,4-dinitrobenzene (CDNB) and 300 μl of supernatant in 2000 μl of total volume. Change in absorbance was recorded at 340 nm. The enzyme activity was calculated as nM CDNB conjugate formed per min/mg protein and the molar extinction coefficient used here was 9.6 × 10^-^^3^ M^−1^ cm^−1^.

#### Reduced glutathione assay (GSH)

Reduced glutathione assay was performed by the method of Jollow et al. [24]. An aliquot of 1000 μl of supernatant of the homogenate was initially precipitated with 1000 μl (4%) of sulfosalicylic acid. After an hour it was centrifuged at 1200×g at 4 °C for 20 min. From the filtrate 100 μl was mixed with 2700 μl of phosphate buffer (pH 7.4; 0.1 M), and 200 μl of 100 mM 1,2-dithio-bis nitro-benzoic acid (DTNB). The absorbance of the reaction mixture was measured immediately at 412 nm. Reduced glutathione activity was determined as μM GSH/g tissue.

#### Lipid peroxidation (TBARS) estimation

Lipid peroxidation assay was executed by following the protocol of Iqbal et al. [25]. The reaction mixture for this assay comprised of 580 μl of phosphate buffer (pH 7.4; 0.1 M), 200 μl of supernatant of the homogenate, 20 μl of ferric chloride (100 mM) and 200 μl of ascorbic acid (100 mM) in a total 1000 μl volume. The incubation of reaction mixture was executed at 37 °C in water bath for an hour. The reaction was stopped by adding 1000 μl of 10% trichloroacetic acid. After the addition of 1000 μl of 0.66% thiobarbituric acid, the conduits were retained for 20 min in boiling water and then placed on ice bath and centrifuged for 10 min at 2500×g. The quantity of TBARS (lipid peroxidation) was calculated by taking the absorbance of supernatant contrary to a reagent blank at 535 nm. The results were articulated as nM TBARS/min/mg tissue at 37 °C using molar extinction coefficient of 1.56 × 10^-^^5^ M^−1^ cm^−1^.

#### Protein estimation

The method of Lowry et al. [26] was followed to estimate total soluble protein of liver tissues by using bovine serum albumin (BSA) standard curve.

#### Hydrogen peroxide assay (H_2_O_2_)

Hydrogen peroxide (H_2_O_2_) assay was performed by following the protocol of Pick and Keisari [27] using the principle of H_2_O_2_-mediated horseradish peroxidase-dependent oxidation of phenol red. The reaction mixture contained 3 ml of tissue homogenate recessed in 1000 μl of 0.28 nM of phenol red, 5.6 nM dextrose, 0.06 M phosphate buffer (pH 7.0) and 8.5 units of horseradish peroxidase and incubated at 37 °C for 60 min. A volume of 10 μl of 10 N of NaOH was added to stop the reaction. After centrifugation for 5 min at 800×g the absorbance of the supernatant was measured at 610 nm against reagent blank. Quantity of H_2_O_2_ produced was given as nM H_2_O_2_/min/mg tissue by using H_2_O_2_ oxidized phenol red standard curve.

#### Nitrite assay

The procedure of Grisham et al. [28] was used to quantify nitrite contents in the samples. After homogenization of the tissue equal volume (100 μl) of 0.3 M NaOH and 5% ZnSO_4_ was mixed for deproteinization. After centrifugation for 15–20 min at 6400×g an aliquot of 30 μl of the supernatant was mixed with 2 ml of Griess reagent in cuvette. The absorbance was recorded at 540 nm. Sodium nitrite curve was used for determining the nitrite amount in tissue samples.

#### Comet assay

DNA mutilation was evaluated by following the protocol of Dhawan et al. [29]. The slides were dipped in methanol then burned over blue flame to get rid of dust and machine oil. About three quarters of sanitized slides were immersed in 1% solution of normal melting point agarose (NMPA) and was endorsed to set at room temperature. A tiny piece of liver tissue was minced into small pieces in 1 ml cold lysing solution. Before coating on already coated slides, the minced tissue was assorted with 85 μl of low melting point agarose solution. The slide was covered gently by placing a cover slip over it and placed for 10–12 min on ice packs. For a second time low melting agarose was added on slide by removing cover slip. Then it was permitted to solidify on ice packs. After coating the slide thrice with low melting agarose, it was retained in lysing solution for 10 min and then in freezer for about 2 h. After performing electrophoresis, staining was done with ethidium bromide (1%) and examined under the fluorescent microscope. The level of DNA damage was appraised by CASP 1.2.3.b software for image analysis. In every sample almost 50–100 cells were examined for head length, comet length, tail moment, tail length and amount of DNA in head of hepatic cell’s nuclei.

#### Histopathological study of tissues

Alterations in histopathology were evaluated via paraffin-embedded staining procedure. The multistep process entails fixation of liver samples in a fixative and were further rinsed and processed in the course of an ascending sequence of alcohol (50, 70, 90 and 100%). The tissues were then secured on hard solid blocks via paraffin-embedding. In the final step, slides were prepared by sectioning 3–4 μm thin layers of the embedded-tissue samples followed by staining with hematoxylin and eosin. Afterwards, these slides were examined under the light microscope (DIALUX 20 EB) at 40X and photographed via HDCE-50B camera.

#### Statistical analysis

The values for all the assays were articulated as mean ± standard deviation. Tukey’s multiple comparison tests centered on parametric scrutiny of variance was used to appraise the enormities of altered treatments to rats in vivo by the use of computer software Statistix 8.1. Statistical significance for demeanors was done at *P*-value ≤0.05.

## Results

### Defensive effect of EDME on liver serum enzymes

The ameliorating effects of EDME against the CCl_4_ induced hepatotoxicity were assessed by determining the level of ALP, AST and ALT in serum of rat (Table 1). Administration of CCl_4_ to rats caused a significant escalation (*p* < 0.05) in the level of ALP = 136.32 ± 4.11 mg/dl, AST = 89.3 ± 3.26 mg/dl and ALT = 173.2 ± 4.19 mg/dl as compared to the ALP = 64.3 ± 3.15 mg/dl, AST = 43.0 ± 2.08 mg/dl and ALT = 44.6 ± 2.22 mg/dl in the control group. The abnormally high level of serum markers was significantly (*p* < 0.05) decreased by co-administration of EDME (200 mg/kg) which tend to regulate these elevated levels (*p* < 0.05) in contrast to that of the control group. However, co-administration of the high dose of EDME (400 mg/kg) significantly decreased the level of these enzymes and the level obtained were as ALP = 71.7 ± 3.39 mg/dl, AST = 53.8 ± 2.33 mg/dl and ALT = 67.4 ± 3.23 mg/dl in serum. The two doses of 200 mg/kg and 400 mg/kg of EDME alone did not induce any alteration (*p* > 0.05) in the level of ALP, AST and ALT of serum as compared to the control group.

**Table 1:** Effect of EDME on biochemical markers of liver in serum

Treatment	ALT (U/I)	ALP (U/I)	AST (U/I)	Serum proteins (mg/dl)	Albumin (mg/dl)	Globulin (mg/dl)
Control	44.6 ± 2.22^e^	64.3 ± 3.15^d^	43.0 ± 2.08^d^	8.5 ± 0.37^a^	5.6 ± 0.2^a^	2.9 ± 0.24^c^
Vehicle Control	44.97 ± 2.16^e^	63.8 ± 2.12^d^	43.3 ± 2.25^d^	8.9 ± 0.40^a^	5.5 ± 0.23^a^	3.0 ± 0.26^bc^
CCl_4_ 1 ml/kg	173.2 ± 4.19^a^	136.3 ± 4.11^a^	89.3 ± 3.26^a^	5.6 ± 0.15^d^	2.1 ± 0.18^d^	3.5 ± 0.16^a^
CCl_4_+ Sily 50 mg/kg	61.8 ± 3.29^d^	71.6 ± 2.41^c^	48.0 ± 1.15^e^	7.5 ± 0.19^b^	4.3 ± 0.19^b^	3.2 ± 0.13^b^
CCl_4_ + EDME 200 mg/kg	104.2 ± 3.21^b^	93.1 ± 3.22^b^	67.1 ± 2.09^b^	6.3 ± 0.22^c^	3.5 ± 0.15^c^	2.8 ± 0.17^c^
CCl_4_ + EDME 400 mg/kg	67.4 ± 3.23^c^	71.7 ± 3.39^c^	53.8 ± 2.33^c^	7.7 ± 0.24^b^	4.7 ± 0.42^b^	3.0 ± 0.18^bc^
EDME 200 mg/kg	46.2 ± 2.16^e^	65.8 ± 3.13^d^	45.3 ± 2.15^d^	8.7 ± 0.16^a^	5.8 ± 0.20^a^	2.9 ± 0.12^c^
EDME 400 mg/kg	45.7 ± 2.31^e^	62.32 ± 3.17^d^	43.3 ± 1.28^d^	8.8 ± 0.21^a^	5.7 ± 0.29^a^	3.1 ± 0.14^bc^

Mean ± SD (*n* = 6), Means with different superscript letters in a column specify significance at *p* < 0.05

*EDME Euphorbia dracunculoides* methanol extract, *Sily* silymarin, *CCl*_*4*_ Carbon tetrachloride

### Defensive effect of EDME on serum protein profile

For the evaluation of protective effects of EDME against the CCl_4_ induced toxicity on liver metabolism the level of albumin, globulin and total serum protein were examined (Table 1). In serum the level of albumin and total protein decreased (*p* < 0.05) while the level of globulin increased with CCl_4_ toxicity in rats as compared to the control group (Table 1). The results showed that EDME have significant protective effects and the altered level of albumin, total protein and globulin induced with CCl_4_ were improved after co-administration of EDME in a dose dependent manner. The protective effects of silymarin for albumin, globulin and total protein were comparable (*p* > 0.05) with the higher dose of EDME 400 mg/kg to rats. Treatment of rats with EDME alone did not (*p* > 0.05) change the level of protein profile as compared to the control group.

### Defensive effect of EDME on liver antioxidant enzymes

Table 2 evaluates the activity level of SOD, CAT, POD and GST in liver homogenates. A significant (*p* < 0.05) decline in the activity of CAT, SOD, POD and GST was observed in liver homogenates of CCl_4_ treated group as compared to the control group. The activity level of CAT, POD, SOD and GST of liver homogenates in CCl_4_ treated group were 1.86 ± 0.12 U/min, 3.93 ± 0.23 U/min, 1.12 ± 0.16 U/mg protein and 8.94 ± 0.92 nM/min/mg protein, respectively. A dose dependent increase in the activity level of CAT, POD, SOD and GST of liver homogenates was recorded with co-administration of EDME. Co-administration of rats with low dose of EDME (200 mg/kg) ameliorated the toxicity of CCl_4_ and increased the activity level of CAT, POD, SOD and GST with values noted as 2.48 ± 0.14 U/min, 4.75 ± 0.35 U/min, 1.78 ± 0.21 U/mg and 16.26 ± 1.07 nM/min/mg protein, respectively. Higher dose of EDME (400 mg/kg) exhibited more protective effects and the activity level recorded were 4.05 ± 0.29 U/min, 6.56 ± 0.72 U/min, 2.46 ± 0.16 U/mg protein and 19.87 ± 2.17 nM/min/mg protein, respectively. Administration of silymarin to CCl_4_ intoxicated rats significantly (*p* < 0.05) increased the level of antioxidant enzyme profile except the SOD as compared to the higher dose of EDME 400 mg/kg administered to rats. However, EDME treatment to rats alone at 400 mg/kg did not affect (*p* > 0.05) the enzymatic level as compared to the of control group.

**Table 2:** Effect of EDME antioxidant enzymes of liver

Treatment	CAT (U/min)	POD (U/min)	SOD (U/mg protein)	GST (nM/min/mg protein)
Control	5.47 ± 0.21^a^	8.66 ± 1.21^a^	3.28 ± 0.17^a^	21.33 ± 2.13^a^
Vehicle control	5.43 ± 0.18^a^	8.61 ± 1.16^a^	3.25 ± 0.28^a^	21.04 ± 2.38^a^
CCl_4_ 1 ml/kg	1.86 ± 0.12^e^	3.93 ± 0.23^e^	1.12 ± 0.16^e^	8.94 ± 0.92^d^
CCl_4_ + Sily 50 mg/kg	4.41 ± 0.26^b^	7.89 ± 1.29^b^	2.73 ± 0.28^b^	21.3 ± 1.33^a^
CCl_4_ + EDME 200 mg/kg	2.48 ± 0.14^d^	4.75 ± 0.35^d^	1.78 ± 0.21^d^	16.26 ± 1.07^c^
CCl_4_ + EDME 400 mg/kg	4.05 ± 0.29^c^	6.56 ± 0.72^c^	2.46 ± 0.16^c^	19.87 ± 2.17^b^
EDME 200 mg/kg	5.46 ± 0.25^a^	8.63 ± 0.92^a^	3.26 ± 0.22^a^	21.32 ± 2.19^a^
EDME 400 mg/kg	5.38 ± 0.32^a^	8.37 ± 0.87^a^	3.31 ± 0.31^a^	21.26 ± 2.45^a^

Mean ± SD (*n* = 6), Means with different superscript letters in a column specify significance at *p* < 0.05

*EDME Euphorbia dracunculoides* methanol extract, *Sily* silymarin, *CCl*_*4*_ Carbon tetrachloride

### Defensive effect of EDME on liver biochemicals

Antioxidant efficacy of EDME was further assessed by determining the concentration of soluble proteins, GSH, TBARS, nitrite and H_2_O_2_ in liver samples of rats (Table 3). Results of CCl_4_ injection to rats showed significant (*p* < 0.05) decrease in the level of soluble protein and GSH in the liver samples as compared to the control group. The co-treatment of EDME with CCl_4_, dose dependently, elevated the protein and GSH content of liver samples in contrary to the group administered with CCl_4_ alone. However, treatment of EDME alone to the rats exhibited non significant (*p* > 0.05) alteration in the protein and GSH content as compared to the control group.

**Table 3:** Effect of EDME biochemical parameters in liver

Treatment	Protein (μg/mg tissue)	GSH (nM//min/mg protein)	TBARS (nM/min/mg protein)	H_2_O_2_ (μg/mg tissue)	Nitrite (μM/ml)
Control	2.55 ± 0.14^a^	19.35 ± 1.18^a^	22.33 ± 2.12^d^	0.23 ± 0.06^e^	50.61 ± 2.13^e^
Vehicle control	2.41 ± 0.05^ab^	19.03 ± 1.11^a^	22.07 ± 2.1^d^	0.24 ± 0.05^e^	50.93 ± 2.15^e^
CCl_4_ 1 ml/kg	0.98 ± 0.08^d^	6.75 ± 0.57^e^	44.52 ± 3.26^a^	0.77 ± 0.19^a^	92.96 ± 3.17^a^
CCl_4_+ Sily 50 mg/kg	2.43 ± 0.23^ab^	18.33 ± 1.08^b^	29.28 ± 2.08^c^	0.27 ± 0.08^d^	55.03 ± 2.07^c^
CCl_4_ + EDME 200 mg/kg	1.66 ± 0.29^c^	9.33 ± 1.18^d^	36.87 ± 2.38^b^	0.58 ± 0.07^b^	73.47 ± 3.2^b^
CCl_4_ + EDME 400 mg/kg	2.11 ± 0.06^b^	14.82 ± 1.33^c^	28.13 ± 2.07^c^	0.44 ± 0.07^c^	60.23 ± 3.24^d^
EDME 200 mg/kg	2.47 ± 0.11^a^	20.24 ± 2.13^a^	23.09 ± 2.2^d^	0.27 ± 0.05^e^	50.62 ± 2.21^e^
EDME 400 mg/kg	2.52 ± 0.16^a^	20.33 ± 1.37^a^	22.67 ± 2.43^d^	0.24 ± 0.03^e^	51.69 ± 2.18^e^

Mean ± SD (*n* = 6), Means with different superscript letters in a column specify significance at *p* < 0.05

*EDME Euphorbia dracunculoides* methanol extract, *Sily* silymarin, *CCl*_*4*_ Carbon tetrachloride

After the completion of treatment, in the liver samples of CCl_4_ intoxicated rats an escalation in the content of TBARS, H_2_O_2_ and nitrite was recorded i.e. 44.52 ± 3.26 nM/min/mg protein, 0.77 ± 0.19 μM/ml and 92.96 ± 3.17 μM/ml, respectively (Table 3). The toxicity of CCl_4_ was ameliorated by the co-administration of EDME in a dose dependent fashion. The level of TBARS, H_2_O_2_ and nitrite in liver samples of rat with co-treatment of the lower dose of EDME 200 mg/kg were; 36.87 ± 2.38 nM/min/mg protein, 0.58 ± 0.07 μM/ml and 73.47 ± 3.2 μM/ml, respectively. The high dose of EDME 400 mg/kg remarkably (*p* < 0.05) lowered the level of TBARS, H_2_O_2_ and nitrite content; 28.13 ± 2.07 nM/min/mg protein, 0.44 ± 0.07 μM/ml and 60.23 ± 3.24 μM/ml, respectively. Moreover, the rats treated with EDME alone did not exhibit alteration in the level of TBARS, H_2_O_2_ and nitrite in liver samples as compared to the control group.

### Comet assay

**Fig. 1:**
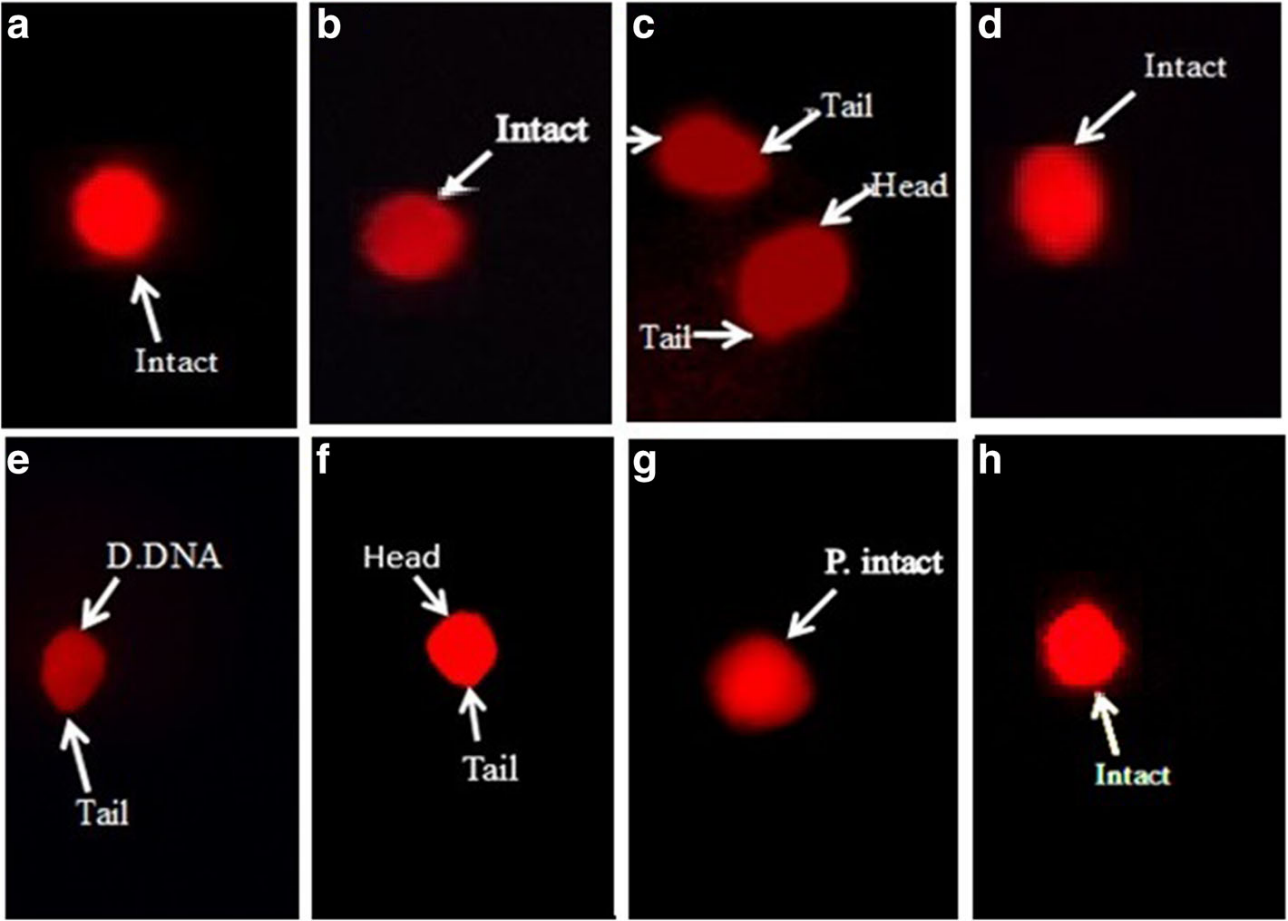
Fluorescence photomicrograph of the protective effects of *E. dracunculoides* methanol extract on DNA damages of liver cells **a** Control group, **b** Vehicle control, **c** CCl_4_ 1 ml/kg, **d** CCl_4_ + Silymarin (50 mg/kg), **e** CCl_4_ + EDME (200 mg/kg), **f** CCl_4_ + EDME (400 mg/kg), **g** EDME (200 mg/kg), **h** EDME (400 mg/kg)

Oxidative stress induced with CCl_4_ in rat displayed extensive DNA damages in the hepatocytes (Fig. 1). Comet parameters such as comet length, tail length, %DNA in tail and tail moment significantly (*p* < 0.05) increased in the hepatocyte of CCl_4_ treated rats as compared to the control group (Table 4). However, head length and %DNA in head decreased in hepatocyte of CCl_4_ treated rats as compared to the control group. In case of CCl_4_ treated rats 22.63% of DNA has migrated from head in the tail of comet. Genotoxicity induced with CCl_4_ in hepatocytes of rat decreased with co-administration of EDME to rats in a dose dependent manner. Administration of EDME 200 mg/kg and 400 mg/kg along with CCl_4_ to rats have displayed potent protective effects and a substantial (*p* < 0.05) decrease in comet length, comet tail, %DNA in tail and tail moment was exhibited in hepatocytes as compared to the CCl_4_ treated group. Further, EDME co-administration to rats increased the head length and %DNA in head of comet of hepatocytes as compared to the CCl_4_ treated group. At lower dose of EDME (200 mg/kg) about 9.75% DNA has migrated while at the higher dose of EDME (400 mg/kg) 5.07% DNA has migrated from head in the tail of comet. Co-administration of silymarin also remarkably (*p* < 0.05) restored the comet parameters in hepatocytes of CCl_4_ intoxicated rats. Migration of DNA from head to tail of comet with silymarin was very low (0.94%). The administration of EDME alone did not cause any migration of DNA from head to tail of comet of hepatocytes.

**Table 4:** Effect of EDME on DNA damages of liver cells by comet assay

Group	Comet length (μm)	Head length (μm)	Tail length (μm)	%DNA in head	%DNA in tail	Tail moment
Control	42.3 ± 1.3^d^	37.6 ± 1.3^a^	4.2 ± 1.2^d^	95.3 ± 1.1^a^	4.13 ± 0.31^d^	0.17 ± 0.08^e^
Vehicle control	41.6 ± 1.94^d^	37.1 ± 0.8^a^	4.87 ± 0.7^d^	94.18 ± 0.9^a^	5.32 ± 0.26^d^	0.25 ± 0.09^e^
CCl_4_ 1 ml/kg	64.5 ± 1.4^a^	23.4 ± 1.2^d^	40.81 ± 0.8^a^	73.9 ± 1.13^d^	25.7 ± 1^a^	10.48 ± 0.6^a^
CCl_4_+ Sily 50 mg/kg	41.9 ± 1^d^	35.2 ± 1.78^b^	6.0 ± 1.19^d^	94.9 ± 0.7^a^	5.03 ± 0.5^d^	0.30 ± 0.07^d^
CCl_4_ + EDME 200 mg/kg	53.7 ± 1.8^b^	32.8 ± 1.4^c^	19.7 ± 1.7^b^	86.5 ± 1.2^c^	13.43 ± 0.7^b^	2.64 ± 0.14^b^
CCl_4_ + EDME 400 mg/kg	48.3 ± 1.21^c^	35.9 ± 1.1^b^	11.63 ± 1^c^	89.6 ± 0.8^b^	10.16 ± 0.9^c^	1.18 ± 0.16^c^
EDME 200 mg/kg	42.5 ± 1.4^d^	37.4 ± 0.9^a^	5.1 ± 0.5^d^	94.92 ± 1.2^a^	5.06 ± 0.8^d^	0.25 ± 0.09^e^
EDME 400 mg/kg	41.1 ± 1.53^d^	37.9 ± 1.87^a^	3.3 ± 0.4^d^	95.71 ± 0.6^a^	4.27 ± 0.5^d^	0.14 ± 0.08^e^

Mean ± SD (*n* = 6), Means with different superscript letters in a column specify significance at *p* < 0.05

*EDME Euphorbia dracunculoides* methanol extract, *Sily* silymarin, *CCl*_*4*_ Carbon tetrachloride

### Defensive effect of EDME on histoarchitecture of liver

**Fig. 2:**
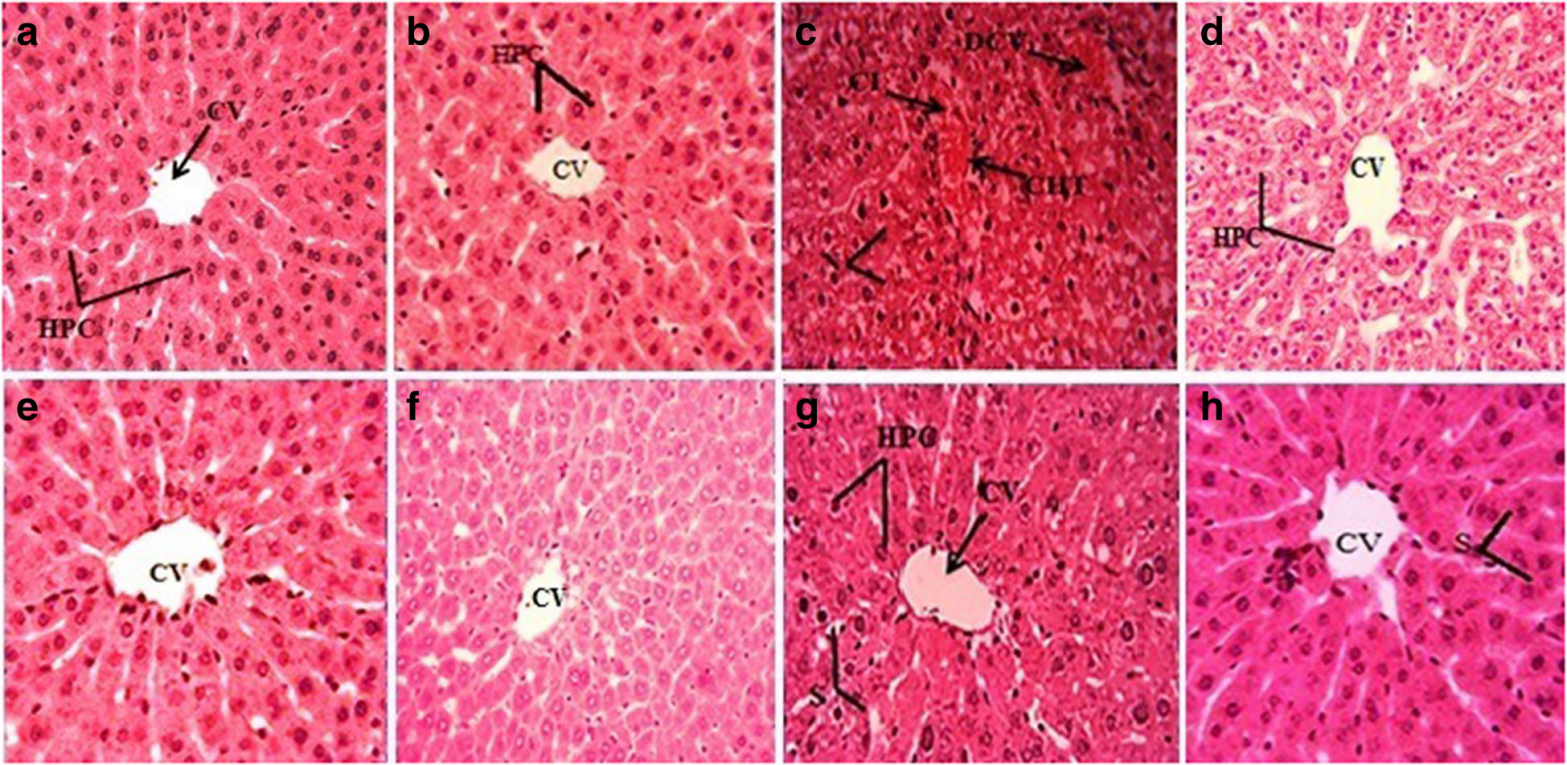
40X Hematoxylin-eosin stain. Histopathological observations for the protective potential of *E. dracunculoides* methanol extract on liver in rat. **a** Control; **b** Vehicle control; **c** CCl_4_ treated; **d** CCl_4_ + Silymarin (50 mg/kg); **e** CCl_4_ + EDME (200 mg/kg); **f** CCl_4_ + EDME (400 mg/kg); **g** EDME (200 mg/kg); **h** EDME (400 mg/kg). CV-Central vein, HPC-Hepatocytes, S-Sinosides, DCV-damaged central vein, N-necrosis, CHT-cellular hypertrophy, CI-Cellular infiltration. EDME- *Euphorbia dracunculoides* methanol extract

The histopathological investigations endorsed protective effects of EDME on the biochemical studies (Fig. 2). The control group displayed normal morphology with typical central vein, kupfer cells, hepatocytes and sinusoids as shown in Fig. 2a and b. Administration of CCl_4_ caused noticeable elevation in fatty changes, inflammatory cells infiltrations, cellular hypertrophy, ballooning, dilation of central vein as shown in Fig. 2c. Treatment with reference drug silymarin (200 mg/kg) attenuated the cellular changes and disruptions as described in Fig. 2d. Administration of low dose of EDME (200 mg/kg) had lessened the hepatic injuries while high dose of EDME (400 mg/kg) potently preserved the typical morphology of liver (Fig. 2e and f). Administration of EDME alone depicted the normal histopatholohy of the liver samples (Fig. 2g and h).

## Discussion

Traditional consumption of plants as medicines is considered non-damaging since centuries. To check the new promising natural antioxidants in plants or to exploit new drug development for tissue related human and animal health, CCl_4_ was used as chemical toxicant for different organs to produce ROS which cause disturbance in the antioxidant system. The current research was conducted to check the defensive prospective of *E. dracunculoides* methanol extract on liver against oxidative stress induced by CCl_4_.

Determination of enzymes such as AST, ALT and AST in the serum provides an important indicator of the hepatic membrane functional integrity. In the present study, elevated levels of liver serum markers ALT, ALP and AST might reflect the hepatic damages due to CCl_4_ intoxication in rats. Enhanced generation of ROS in hepatocytes lead to cellular death due to DNA damage, protein oxidation, lipid peroxidation and also added to the hepatic damaging action [30]. Co-administration of silymarin along with CCl_4_ displayed the protective ability and the values of ALT, ALP and AST were found near to the level of control group. The methanol extract of plant showed its protective aptitude against the CCl_4_ induced hepatotoxicity in rat in a dose dependent fashion. The lower dose (200 mg/kg) of EDME exerted protective aptitude and the lower level of ALT, ALP and AST was recorded whereas the higher dose of EDME (400 mg/kg) exhibited potent toxico-suppressive effects and the level of above parameters was found near to the level of control animals. The elevated level of ALP, AST and ALT in serum with CCl_4_ treatment might be associated with membrane damage of liver cells. Restoration of the hepatic enzymes towards the control level with EDME indicated the hepatoprotective potential of the extract [30, 31].

On the other hand a marked decline in the level of total proteins and albumin while increase of globulin in serum of CCl_4_ treated group was determined in this study. Presence of albumin and globulin in normal range is essential for the proper physiology of a person. Globulin helps to regulate the function of the circulatory system. Globulins consist of four proteins; alpha-1, alpha-2, beta and gamma globulin and among them liver produces predominantly the alpha and beta globulins. Increase in the level of globulin in serum might be due to the prolong treatment of CCl_4_ to rat. Elevated level of globulin in serum can be considered a reliable non-invasive marker towards the degree of liver fibrosis [32]. Co-treatment with low dose of the EDME (200 mg/kg) was protective and revert the levels of serum proteins towards the control samples while the higher dose (400 mg/kg) showed more promising results and restored serum proteins to levels that are analogous to the control group. The protective abilities of EDME might be attributed by the presence of antioxidant phyto-constituents [19].

Antioxidant defense system includes the enzymatic antioxidants (CAT, POD, SOD, GST and GSH) which have vital role in defense system. Catalase (CAT) is a very important enzyme which can neutralize H_2_O_2_ through catalytic conversion to H_2_O and O_2_ at higher concentrations or through peroxidation activity at lower concentration. SOD enzyme dismutates the superoxide anion radical to less toxic O_2_ and H_2_O_2_ by oxidation reduction mechanism of its active metal ion. Glutathione peroxidase (POD) is involved in the catalytic reduction of H_2_O_2_ and lipid peroxides at the expenditure of glutathione. GSH is a free radical scavenger and shielded the toxic effects of various peroxides. During oxidative stress the level of these antioxidants is compromised. In the present study, CCl_4_ injection to rats showed the reduced levels of CAT, POD, SOD, GST and GSH. This condition might reflect the excessive generation of ROS which inhibit the synthesis of antioxidant enzymes. Decreased level of GSH might be due to its more consumption by the hepatocytes in scavenging toxic radicals generated by CCl_4_. Younis et al. [33] also reported the reduced levels of all above enzymes and GSH content in liver tissue by CCl_4_ administration. The antioxidant effects of EDME might improves the levels of antioxidant enzymes including CAT, POD, SOD and GST, and GSH that was declined due to CCl_4_ action. Presence of flavonoids, tannins, sterols, terpenoids might be suspected for the attribution of antioxidant activities [19]. The same study indicated the presence of strong antioxidant phyto-constituents i.e. rutin, myricetin, catechin and caffeic acid in *E. dracunculoides* [19]. Similar protective effects comments were also reported by using *Alnus nitida* against CCl_4_ induced oxidative stress in liver [34]. Presence of various polyphenols in the extract might be responsible for the scavenging of free radicals and in the inhibition of lipid peroxidation [35]. Different studies have also reported protective effects of various plant extracts against the CCl_4_ induced hepatic damages in rat [17, 18, 31].

CCl_4_ also exerts its toxic effects by changing the level of TBARS, H_2_O_2_, tissue protein and nitrite content. In CCl_4_ intoxication the tissue protein level decreases but there is noteworthy increase in the lipid peroxidation (TBARS), H_2_O_2_ and nitrite content. Concentration and duration of CCl_4_ treatments to experimental animals exhibit varied pattern of oxidative injuries. Administration of CCl_4_ for short duration to rat regulates the endogenous expression of interleukin (IL)-6 and IL-10 which in turn suppresses the synthesis of tumor necrosis factor-β1 (TGF-β1) and consequently inhibits the synthesis of collagen. However, longer duration of the treatment of mice with CCl_4_ causes the infiltration of neutrophils and fibrosis of liver [36, 37]. Parola and Robino [38] elaborated that peroxidation of lipids induce overexpression of fibrogenic cytokines by stimulating the synthesis of collagen and activating hepatic stellate cells. It might be assumed that that long term administration of CCl_4_ to rats might increase the oxidative stress and consequently the hepatic fibrosis. In this study hepatic histopathology of rats treated with CCl_4_ exhibited macro steatosis in hepatocytes of rat. Co-administration of silymarin to rats diminished the CCl_4_ toxic effects and showed the level of TBARS, H_2_O_2_ and nitrite content of liver near to the level of control rats. Administration of EDME to CCl_4_ intoxicated rats decrease the level of TBARS, H_2_O_2_ and nitrite content of liver homogenates and the level of above parameters lies near the level of control group. Our findings are supported by the work of Sajid et al. [34] who reported the in vivo antioxidant effects of *Alnus nitida* in rat liver intoxicated with CCl_4_.

Oxidative stress induced with CCl_4_ in liver causes the single stranded or the double stranded break in the DNA and damages the DNA integrity [39]. In the present study, comet assay was performed to check the DNA damage in terms of comet length, head length, %DNA in head, tail length, %DNA in tail and tail moment. A noteworthy elevation in comet length, tail length, tail moment while sharp decline in the level of DNA in head of comet was observed in the hepatocyte of CCl_4_ intoxicated rats in comparison to control group. DNA damages induced with CCl_4_ toxicity in liver has tremendously contributed towards the migration of DNA (22.63%) from head to the tail that consequently resulted in the increase of tail length, %DNA in tail and in tail moment of hepatocytes [39]. Oxidative damage caused by CCl_4_ to DNA in the hepatic cells of rat has also been reported in previous study [39]. Co-administration of EDME, dose dependently, lessened the toxic effects of CCl_4_ on DNA damages. Co-administration of EDME causes an increase in head length and %DNA in head of comet along with a decrease in the mean values of comet length, tail length, tail moment and %DNA in tail suggesting the protective abilities of EDME against the CCl_4_ induced DNA damages. Treatment of EDME to CCl_4_ intoxicated rats substantially inhibited the migration of DNA from head to tail in comet of hepatocytes. At lower dose of EDME (200 mg/kg) there was 9.75% DNA migration from head to tail while 5.07% DNA migration from head to tail of comet was recorded at higher dose of EDME (400 mg/kg) suggesting the toxico-suppressive effect of EDME.

Number of DNA strand breaks (tail moment −1) provides a useful estimate for the protective abilities of the extract. Generation of ROS is considered to be involved in the oxidative DNA damages. In this investigation DNA strand breaks were not determined in the control group as well as the groups treated with EDME alone at both 200 mg/kg and 400 mg/kg treatment to rats. However, treatment of CCl_4_ to rats caused 9.48 number of DNA strand breaks in the comet of hepatocytes. The co-administration of EDME to CCl_4_ treated rats decreased the number of DNA stand breaks; 1.64 (200 mg/kg) and 0.181 (400 mg/kg) of EDME, respectively. DNA strand breaks were not recorded with the co-administration of silymarin to CCl_4_ intoxicated rats. These results suggested that EDME is effective in decreasing the ROS level, DNA fragmentation and in increasing the cell viability. DNA damaging effects of CCl_4_ might be ameliorated by the various phytochemical classes reported in *E. dracunculoides* [19]. Protection of DNA damages induced with hydrogen peroxide in human lymphocytes has been demonstrated with various herbal extracts [40].

The histology of the liver is the direct mean of assessing the protective effects of therapeutic agents against CCl_4_ induced hepatic damages. CCl_4_ induced the high degree of damage in liver cells, by inducing fibrosis, necrosis, cellular hypertrophy and central lobule disruption. Co-administration of silymarin as well as low and high dose of EDME ameliorated the toxic effects of CCl_4_ and decreased the damages induced with CCl_4_ intoxication. The results of this study are in accordance with previous reports [41]. The protective effects exhibited by the EDME might be attributed by the presence of flavonoids, terpenoids, sterols and tannins. *E. dracunculoides* was found to be constituted by rutin, catechin, myricetin and caffeic acid [19].

## Conclusions

Our study recommends that *E. dracunculoides* have the aptitude to ameliorate the hepatic damage provoked by CCl_4_ and has potential to restore the levels of enzyme activity, serum markers, DNA damages and histopathological amendments. The defensive properties of EDME might probably be concomitant with its phytochemical profile and antioxidant properties.

## Abbreviations

ALP: Alkaline phosphatase
ALT: Alanine transaminase
AST: Aspartate transaminase
CAT: Catalase
EDME: *Euphorbia dracunculoides* methanol extract
GSH: Reduced glutathione
GST: Glutathione-S-transferase
POD: Peroxidase
SOD: Superoxide dismutase
TBARS: Thiobarbituric acid reactive substances
